# Technology overload, job crafting, and work engagement: the moderating role of mindfulness

**DOI:** 10.3389/fpsyg.2026.1842144

**Published:** 2026-07-23

**Authors:** Xin Li, Qing Tian, Fang Pei

**Affiliations:** 1 School of Economics and Management, Civil Aviation Flight University of China, Guanghan, China; 2 School of Business, Macau University of Science and Technology, Taipa, Macao SAR, China

**Keywords:** challenge-hindrance demands framework, conservation of resources theory, energy depletion, job crafting, technology overload, transactional stress appraisal, work engagement

## Abstract

**Introduction:**

Employees in digitally mediated workplaces may experience technology overload (TO) when digital information, communication demands, and system features exceed their capacity to respond. Although TO is generally associated with strain, prior findings are mixed, suggesting that employees’ appraisals of digital demands and their subsequent work adjustments may shape its consequences. Guided by the Challenge–Hindrance Demands Framework and Conservation of Resources theory, with stress appraisal treated as the linking process, this study examined the indirect association between TO and work engagement and the role of mindfulness in this process.

**Methods:**

Three-wave survey data were collected from 438 employees in Chinese organizations. The analysis tested a sequential indirect pathway from TO to work engagement through energy depletion and job crafting. It also examined whether mindfulness altered the strength of these associations.

**Results:**

TO was positively associated with subsequent energy depletion. Greater depletion was, in turn, associated with more job crafting, which was positively related to work engagement. The direct association between TO and work engagement was not statistically significant. Mindfulness more clearly strengthened the association between TO and energy depletion, a pattern consistent with greater awareness of strain. Its association with adaptive responses following depletion was also positive, but the evidence was weaker.

**Discussion:**

The findings do not suggest that TO is beneficial or sustainable. Instead, they indicate that employees may try to adapt to overload by changing aspects of their work, particularly when they recognize the strain and retain sufficient regulatory resources to respond.

## 1 Introduction

Digital technology is now integrated into daily workflows, changing the way employees coordinate tasks and communicate with each other. The empirical context of this study is digitally mediated work, where employees routinely use enterprise information systems, communication and collaboration platforms, data-driven applications, and other digital tools to complete and coordinate work. These tools are often adopted to improve operational efficiency and reduce costs (Wang and Shao, 2024), but they may also require employees to manage multiple systems, respond to messages in real time, and remain available online. Such demands increase cognitive and time pressure, speed up the pace of work (Tarafdar et al., 2015), and blur the boundaries between work and personal life (Tarafdar et al., 2018). They also help explain the growing attention to technology overload (TO), a perceived technology-related pressure that arises when continuous interaction with digital tools exceeds employees’ cognitive and temporal resources (Ragu-Nathan et al., 2008; Karr-Wisniewski and Lu, 2010; Rasool et al., 2022; Castillo et al., 2023).

Much technostress research has treated TO mainly as a harmful demand, emphasizing its negative implications for employees and organizations (Karr-Wisniewski and Lu, 2010; Delpechitre et al., 2018). More recent work suggests that this view may be incomplete. Employees may appraise TO differently depending on digital demand intensity and available resources, such as technical support, training, participation in technology-related changes, and psychological safety (Lazarus and Folkman, 1984; Ragu-Nathan et al., 2008; Zhao et al., 2020; Wang and Yao, 2023; Lim et al., 2025). Recent validation work also suggests that technostress creators should be interpreted alongside inhibitors that help employees manage them (Castillo et al., 2023). TO can require employees to learn, adapt, and build new capabilities while still creating strain (Califf et al., 2020; Yu et al., 2023). Employees may also use adaptive strategies, including skill development and job crafting, despite the strain produced by overload (Ingusci et al., 2021). These findings suggest that employees do not only passively experience technology-related demands; they may also adjust their work to remain engaged. However, it remains unclear how employees move from the resource costs of TO to adaptive responses, and whether these responses can translate into higher work engagement.

Cultural background may also be relevant to understanding responses to TO. Much of the available evidence comes from Western contexts, leaving open the question of how these processes unfold in other environments. This study focuses on Chinese organizations, where strong performance expectations and hierarchical structures are common. These characteristics may make job demands more likely to be interpreted as opportunities to demonstrate ability or gain recognition (Gaan, 2016; Gabel-Shemueli et al., 2019). This environment provides a relevant context for studying how employees respond to TO.

This suggests that TO differs from general workload or time pressure. It reflects employees’ perceived overload arising from digitally mediated work demands. It may function as a challenge-like demand only when employees have sufficient support, opportunities to adapt, and some discretion in managing these demands (Lazarus and Folkman, 1984; Ragu-Nathan et al., 2008; Zhao et al., 2020; Castillo et al., 2023). The Challenge-Hindrance Framework (CHDF) captures the possibility that demands may be interpreted as challenges or hindrances, but it provides limited insight into how employees arrive at such appraisals (Podsakoff et al., 2023). The transactional model of stress suggests that stress responses depend on how employees appraise a situation and the resources they believe they have for dealing with it (Lazarus and Folkman, 1984). In addition, the job demand-control perspective suggests that high demands are more likely to produce strain when decision latitude is low (Karasek, 1979). The Conservation of Resources (COR) theory provides a complementary perspective, focusing on how individuals manage resources under pressure. Investing resources without sufficient recovery may lead to energy depletion, whereas adjusting job demands or mobilizing resources may help individuals adapt (Hobfoll, 1989, 2002; Hobfoll et al., 2018). Therefore, TO may lead to withdrawal, health risks, or adaptive engagement depending on employees’ appraisals and the resources they have or can access.

Even if TO is appraised as challenge-like, it can still lead to energy depletion. Continuous resource investment without sufficient recovery may promote a loss spiral (Hobfoll et al., 2018). Frequent task switching and fragmented communication increase the need for cognitive effort and self-regulation. If employees cannot effectively manage stress in a TO environment, they are more likely to experience energy depletion (Bartek et al., 2023). This depletion should therefore be understood as resource loss, not as a desirable motivational mechanism. Repeated exhaustion and insufficient recovery can undermine employees’ long-term well-being and work functioning (Maslach et al., 2001; Schaufeli et al., 2002).

When employees’ energy is depleted, they may respond differently. Some individuals withdraw from their work, while others readjust their working styles. Job crafting reflects a more active response, including adjusting tasks or working conditions to manage demands and maintain work status (Wrzesniewski and Dutton, 2001; Zhang and Parker, 2019). However, not all employees engage in such adjustments (Petrou et al., 2015). When job crafting occurs, it may help maintain work engagement (Bakker and Oerlemans, 2019). This raises an important question: under what conditions and how can energy depletion be transformed into constructive adjustments?

Mindfulness has been identified as one such condition, with evidence showing that it may reduce psychological stress and emotional exhaustion at work (Brown and Ryan, 2003; Hülsheger et al., 2013). Mindfulness involves awareness of internal states and sustained attention, which may help employees maintain focus under continuous job demands and regulate their responses in TO-intensive environments (Reb et al., 2015; Ioannou, 2023). Studies have linked mindfulness to lower perceived technostress, cognitive strain, and burnout-related outcomes, although its effects may depend on the specific work context (Hülsheger et al., 2013; Ioannou, 2023). Mindfulness may not operate only as a stress-buffering resource. Heightened monitoring of present experiences may also make demanding work conditions and internal signs of strain more salient, especially when awareness is not accompanied by acceptance or when the work context is adverse (Lindsay and Creswell, 2017; Walsh and Arnold, 2020; Lyddy et al., 2021). Mindfulness may therefore have different implications across the resource adaptation process. During exposure to TO, it may shape how employees notice and interpret resource demands; after depletion occurs, it may support more deliberate regulation of subsequent behavior. This reasoning is consistent with Monitor and Acceptance Theory (MAT), which distinguishes monitoring, a process that increases awareness of present-moment experiences, from acceptance, a process that supports adaptive regulation of those experiences (Lindsay and Creswell, 2017).

This stage-specific role is particularly important in the context of TO, where employees must respond to continuous digital demands while maintaining work engagement. Previous studies have mainly focused on its negative effects (Karr-Wisniewski and Lu, 2010; Delpechitre et al., 2018), with less attention paid to how employees respond to digital strain in their daily work. Thus, the key issue is not whether technology overload is harmful or beneficial in itself, but how employees appraise and manage strain when digital work consumes psychological, temporal, and attentional resources. This raises an important question of when energy depletion leads to constructive work adjustments rather than withdrawal, and how mindfulness shapes this process.

Drawing on the CHDF and COR theory, this study examines how employees may maintain work engagement through resource-based adaptation after experiencing technology overload. TO may consume employees’ energy by increasing cognitive and self-regulation demands. However, this depletion does not necessarily lead only to work withdrawal. Employees may also engage in job crafting by adjusting their tasks, relationships, or work boundaries. These adjustments may help them regain a sense of control and maintain work functioning.

Previous research has mainly treated TO as a source of stress, impaired well-being, or reduced performance (Karr-Wisniewski and Lu, 2010; Tarafdar et al., 2015; Delpechitre et al., 2018). Recent studies have further suggested that employees may respond to TO by developing new skills or crafting their jobs (Califf et al., 2020; Ingusci et al., 2021; Yu et al., 2023). However, it remains unclear how employees move from energy depletion to adaptive job crafting, and whether this process helps sustain work engagement without obscuring the negative health implications of depletion. Mindfulness is also considered a boundary condition, since it may shape how employees perceive digital stress and regulate their subsequent behavior (Brown and Ryan, 2003; Lindsay and Creswell, 2017). In Chinese organizations, performance expectations and responsibility norms may influence how employees understand work demands (Hofstede, 2011; Tran et al., 2022). Thus, this study offers a more context-sensitive explanation of the relationships among TO, energy depletion, job crafting, and work engagement.

## 2 Theoretical background

### 2.1 Challenge-hindrance demands and the conservation of resources

The CHDF classifies job demands according to how they are appraised, distinguishing challenge demands that may promote personal growth and achievement from hindrance demands that obstruct goal attainment (Lepine et al., 2005; Crawford et al., 2010). However, whether a demand is experienced as a challenge, hindrance, or threat is not determined by the demand alone. The transactional model of stress emphasizes that employees appraise what is at stake and whether they have sufficient resources to manage the demands (Lazarus and Folkman, 1984). When there are sufficient work resources and decision latitude, despite the intensity of demands such as workload or time pressure, these demands may still be regarded as opportunities for learning and development (Karasek, 1979; Podsakoff et al., 2007; Van Den Broeck et al., 2010). COR theory further expands this idea, focusing on how individuals allocate, preserve, and mobilize resources under pressure to maintain engagement (Hobfoll, 1989; Hobfoll et al., 2018). Together, these frameworks show that employee responses are influenced by demand appraisal, perceived resource availability, and the degree of control employees have over how work is performed.

TO shares some characteristics with challenge demands because it requires substantial cognitive and time investment. Yet TO is not simply the digital demand itself. In this study, TO is understood as employees’ perceived overload from digital work demands, which are shaped by the systems they use, the expectations attached to those systems, and the everyday activities through which work is carried out. This view is consistent with work-activity research showing that digital technologies can reshape how tasks are organized, interrupted, and monitored, influencing both prescribed work and the activity employees actually perform (Bobillier-Chaumon, 2021). Whether TO is experienced as challenge-like therefore depends on the conditions under which employees manage digital demands. With usable support and sufficient discretion, employees may respond by improving digital skills, prioritizing information, or reorganizing work processes. Prior research suggests that adaptive responses to TO, including timely information processing and work-control support, are associated with job satisfaction and psychological adaptability (Yin et al., 2018). This points to the value of adaptive responses to overload, not the inherent value of overload itself.

This appraisal-based perspective offers a useful lens for understanding technostress, as different techno-stressors may be appraised differently. TO, in particular, may be appraised as challenging when employees view increased digital workload as requiring effort, learning, and adaptation rather than simply as an obstacle (Zhao et al., 2020; Wang and Yao, 2023; Lim et al., 2025). The present study therefore does not treat TO as inherently beneficial or as a challenge demand under all conditions. Instead, TO is conceptualized as a perceived digital overload state that involves resource costs and may be followed by adaptive responses only when employees have sufficient resources, control, and organizational support.

### 2.2 Theoretical model and hypotheses

Building on the CHDF, COR theory, and transactional stress appraisal, we propose a sequential model in which TO, understood as a perceived overload state with high cognitive and time requirements, leads to energy depletion. To restore resource balance, employees may engage in compensatory job crafting behaviors, which in turn support the maintenance of work engagement. We further propose that mindfulness, as a personal resource, shapes this process by affecting the impact of TO on energy depletion and the link between depletion and job crafting. The model outlines how employees can respond to digital demands through continuous psychological and behavioral adaptation while recognizing that depletion itself remains a costly and potentially harmful state.

#### 2.2.1 TO and energy depletion

Energy depletion refers to the loss of psychological energy available after continuous self-control efforts, which consume cognitive and emotional resources, especially when work becomes heavy or strictly controlled (Ryan and Deci, 2008). Challenge demands may contribute to development, but they still require sustained efforts, and if there is not enough recovery, these efforts may accumulate into pressure (Lepine et al., 2005; Podsakoff et al., 2007). TO is conceptualized as a continuous working state in which employees constantly respond to cross-platform information, manage large amounts of digital information, and adapt to new systems as deadlines approach. There is little opportunity to disengage from work (Califf et al., 2020). These demands continue to put pressure on attention and self-regulation resources, limiting opportunities for recovery, and are more likely to lead to fatigue and psychological exhaustion over time (Xanthopoulou et al., 2007). In this sense, energy depletion is closer to the exhaustion component emphasized in burnout and engagement research than to a positive motivational state (Maslach et al., 2001; Schaufeli et al., 2002).

COR theory explains that stress not only comes from the loss of resources, but also from continuous investment with limited returns (Hobfoll, 1989, 2002, 2011). In the performance-oriented environment of many Chinese organizations, recovery behaviors such as temporarily leaving work may be interpreted as insufficient investment (Hu et al., 2014), which makes it difficult for employees to reduce investment even when resources are tight. Therefore, even when experiencing strain, employees may continue to engage in their work, gradually accelerating energy consumption. When opportunities for recovery are limited, TO may still involve motivational potential while consuming substantial resources (Lepine et al., 2005). As energy is depleted, this indicates the need to conserve remaining resources or adjust engagement to maintain work performance. Accordingly, TO is expected to be positively related to energy depletion. Based on this, we propose the following hypothesis.

Hypothesis 1 (H1): TO is positively associated with energy depletion.

### 2.3 Energy depletion and job crafting

Job crafting refers to employees’ self-initiated adjustments to the task, relational, and cognitive aspects of work, aimed at improving person-job fit (Wrzesniewski and Dutton, 2001; Zhang and Parker, 2019). In this study, job crafting is treated as a form of self-regulation through which employees adjust job demands and job resources. This view aligns with the job crafting scale adopted here, which captures increases in structural and social resources, increases in challenging demands, and reductions in hindering demands (Tims et al., 2012). Job crafting is not confined to proactive expansion when resources are sufficient. It can also help employees align work demands and resources with their needs, abilities, and goals when resources are constrained (Tims et al., 2012; Wang et al., 2018; Lim et al., 2025). Job crafting, therefore, provides a mechanism through which employees can adjust job demands and resources when work pressure creates a resource imbalance.

Drawing on the CHDF, challenge-like demands can involve both resource costs and possible adaptive responses. They require sustained effort and may deplete employees’ energy, while also signaling that current ways of working need to be adjusted (Lepine et al., 2005; Crawford et al., 2010). Under challenge-like demands, energy depletion may indicate that current work practices are no longer sustainable. Employees may then need to reconsider how they allocate effort, manage demands, and access resources. This does not make depletion desirable. It may instead serve as a warning signal that prompts adjustment when resources and decision latitude are available.

COR theory further explains why employees may still engage in adjustment after depletion. When resources are threatened or lost, individuals may conserve remaining resources and invest selectively to limit further loss or rebuild resources (Halbesleben et al., 2014; Hobfoll et al., 2018). Prior studies suggest that job crafting may occur when employees perceive a mismatch between role expectations and available resources (Tims et al., 2013; Kuijpers et al., 2020), and that it can function as a compensatory mechanism under demanding conditions (Petrou et al., 2015; Hakanen et al., 2018; Wang et al., 2018; Doden et al., 2024). These arguments suggest that energy depletion may make job crafting more likely when employees continue to face challenge-like demands.

This reasoning applies closely to TO because it requires employees to handle increased digital workload, frequent ICT-mediated communication, and time-pressured information processing, which may consume cognitive, temporal, and self-regulatory resources (Ragu-Nathan et al., 2008; Ayyagari et al., 2011). Recent technostress research further suggests that employees do not appraise all technology-related demands in the same way. TO, in particular, has been found to be more closely associated with challenge appraisal outcomes than some other techno-stressors, such as techno-complexity or techno-invasion (Zhao et al., 2020; Wang and Yao, 2023). This is consistent with the transactional view that coping depends on appraisal and available resources (Lazarus and Folkman, 1984). When TO is appraised as challenge-like and employees have resources to act, they may respond to technology-related demands through coping and adaptation rather than withdrawal alone. Such responses may include developing IT capacity, exercising greater control over technology use, or positively reinterpreting technology-related demands to reduce their effects on IT-enabled productivity and work functioning (Beaudry and Pinsonneault, 2005; Tarafdar et al., 2015; Pirkkalainen et al., 2019). Empirical studies also link TO and related techno-stressors to job crafting under certain conditions, including remote work and psychologically safe contexts (Ingusci et al., 2021; Philip and Kosmidou, 2023; Lim et al., 2025).

Energy depletion under TO may signal a mismatch between digital work demands and available resources. Employees may respond by reorganizing task priorities, seeking feedback or support, or reducing hindering demands. However, this response depends on more than individual willingness. Job crafting is more plausible when employees have autonomy, decision latitude, and organizational flexibility to modify how digital work is organized (Karasek, 1979; Tims et al., 2012). Even when formal flexibility is limited, employees may still adjust how they perform their work, especially when these adjustments help them remain aligned with organizational expectations (Wrzesniewski and Dutton, 2001; Bakker and Oerlemans, 2019). We therefore expect energy depletion to be positively associated with job crafting, while recognizing that this relationship should be interpreted as a conditional adaptive response rather than as evidence that depletion is desirable. Combined with H1, this expected association suggests an indirect relationship between TO and job crafting through energy depletion. We propose the following hypothesis.

Hypothesis 2 (H2): Energy depletion is positively associated with job crafting.

Hypothesis 3 (H3): Energy depletion mediates the relationship between TO and job crafting.

### 2.4 Job crafting and work engagement

Job crafting is closely related to work engagement because it helps employees bring their work closer to their needs and goals (Demerouti et al., 2015; Zhang and Parker, 2019). By adjusting job demands and resources, employees may gain greater autonomy and access to feedback or support, which can sustain vigor, dedication, and absorption at work (Wrzesniewski and Dutton, 2001; Bakker et al., 2012; Tims et al., 2013). From the COR perspective, these adjustments can also be understood as resource investment behaviors through which employees protect or rebuild job resources under pressure (Hobfoll et al., 2018). Prior research generally shows that approach-oriented forms of job crafting, especially seeking resources and challenges, are positively associated with work engagement, whereas demand-reducing forms may have weaker or more mixed effects (Tims et al., 2013; Petrou et al., 2015; Rudolph et al., 2017; Zhang and Parker, 2019). In the present study, job crafting is treated as a broad form of active work adjustment. Some forms may be more strongly tied to engagement than others, but each involves employees’ efforts to reshape work conditions in ways that help preserve resources and sustain task involvement. We therefore expect job crafting to be positively associated with work engagement.

H2 proposes that energy depletion is positively associated with job crafting. This crafting response is theoretically meaningful because it offers a behavioral means through which depleted employees can reorganize work demands and resources instead of only experiencing lower engagement. When such adjustments help employees regain control by bringing work demands closer to their needs and available resources, job crafting can transmit the relationship between energy depletion and work engagement. We therefore propose the following mediation hypothesis:

Hypothesis 4 (H4): Job crafting is positively associated with work engagement.

Hypothesis 5 (H5): Job crafting mediates the relationship between energy depletion and work engagement.

#### 2.4.1 Mindfulness as a moderator

Mindfulness refers to a self-regulatory capacity involving sustained attention to present experiences and non-judgmental awareness of internal states (Brown and Ryan, 2003). Prior research has linked mindfulness to better emotion regulation and attention control (Glomb et al., 2011), as well as lower psychological stress and emotional exhaustion at work (Hülsheger et al., 2013; Reb et al., 2015; Ioannou, 2023). However, these effects may depend on how mindfulness interacts with employees’ self-regulation and the demands they face at work (Walsh and Arnold, 2020; Lyddy et al., 2021; Krick et al., 2023). TO requires employees to process excessive information, respond to frequent interruptions, and switch rapidly between tasks (Grandhi et al., 2005; Tarafdar et al., 2007; Ayyagari et al., 2011). Although mindfulness has received growing attention, less is known about how it shapes employees’ experience of TO and their subsequent responses to energy depletion. MAT helps explain this dual role of mindfulness: monitoring increases awareness of internal signals and situational demands, whereas acceptance helps individuals regulate these experiences without avoidance or overreaction (Lindsay and Creswell, 2017).

As a self-regulatory resource, mindfulness may affect both what employees notice under TO and how they respond to depletion. TO creates the resource demands, while mindfulness may make employees more aware of their costs. Employees with higher mindfulness may notice signs of strain more readily because they are more attentive to present experiences and internal states (Lindsay and Creswell, 2017). As TO increases, this awareness may make accumulated fatigue and sustained attentional effort more consciously experienced. Prior research similarly suggests that mindfulness can heighten awareness of demanding work conditions and internal strain signals, rather than simply buffering them (Walsh and Arnold, 2020; Lyddy et al., 2021). We therefore expect mindfulness to strengthen the relationship between TO and experienced energy depletion. This reasoning leads to the following moderation hypothesis:

Hypothesis 6a (H6a): Mindfulness moderates the positive relationship between TO and energy depletion, such that the relationship is stronger when mindfulness is higher.

Employees who become aware of energy depletion still need to decide how to respond. Recognizing depletion does not automatically lead to job crafting. Mindfulness may affect this next step because it is associated with stronger self-regulation and lower avoidance under stress (Weinstein et al., 2009; Donald and Atkins, 2016; Cao et al., 2022). From a COR perspective, these tendencies can help employees protect or restore valuable internal resources under strain (Hobfoll et al., 2018). Related work links mindfulness to recovery-oriented self-regulation during exhaustion (Dvořáková et al., 2019), and suggests that employees under strain may use role adjustment or compensatory strategies as adaptive responses to depletion (Hur et al., 2024). Mindful employees may therefore be more likely to respond through job crafting, adjusting demands, or securing support to conserve resources and maintain task involvement. Accordingly, we propose the following moderation hypothesis:

Hypothesis 6b (H6b): Mindfulness moderates the positive relationship between energy depletion and job crafting, such that the relationship is stronger when mindfulness is higher.

These moderation arguments also extend to the indirect paths in the model. If mindfulness strengthens the link between TO and experienced energy depletion, the indirect relationship between TO and job crafting through energy depletion should be stronger among employees with higher mindfulness. Similarly, if mindfulness helps depleted employees respond through deliberate job crafting, the indirect relationship between energy depletion and work engagement through job crafting should also be stronger when mindfulness is higher. Thus, we propose the following moderated mediation hypotheses.

Hypothesis 7a (H7a): Mindfulness moderates the indirect relationship between TO and job crafting through energy depletion, such that this indirect relationship is stronger when mindfulness is higher.

Hypothesis 7b (H7b): Mindfulness moderates the indirect relationship between energy depletion and work engagement through job crafting, such that this indirect relationship is stronger when mindfulness is higher.

**Figure 1:**
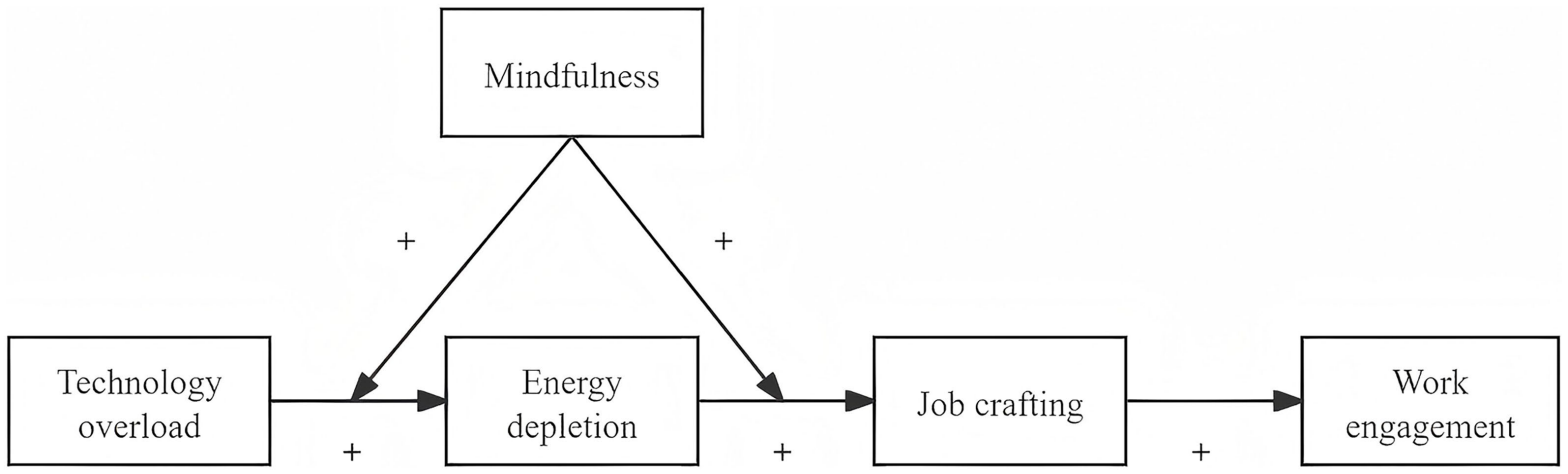
The proposed moderated mediation model. Mindfulness moderates the direct effects of technology overload on energy depletion (H6a) and of energy depletion on job crafting (H6b), as well as the corresponding indirect effects (H7a and H7b).

The proposed theoretical model is shown in Figure 1.

## 3 Methods

### 3.1 Measurement

All variables were measured using previously validated scales. Unless otherwise stated, items were rated on a 5-point Likert scale ranging from 1 (strongly disagree) to 5 (strongly agree). Scale scores were computed by averaging the relevant items after applying the original coding guidelines.

Technology overload was measured using the retained 11-item scale adapted from Karr-Wisniewski and Lu (2010), covering information overload (3 items, Cronbach’s *α* = 0.803), communication overload (4 items, Cronbach’s α = 0.778), and system feature overload (4 items, Cronbach’s α = 0.846). A sample item is “I am often distracted by the excessive amount of information available to me for business decision-making.” This operationalization captures employees’ perceived overload from information volume, communication demands, and system complexity in digital work, not simply the number of technologies they use (Castillo et al., 2023). The overall scale showed high internal consistency (Cronbach’s α = 0.903).

Energy depletion was assessed using four reverse-coded items adapted from Ryan and Frederick’s (1997) Individual-Difference Level Subjective Vitality Scale, following Deng et al. (2018), rather than the State Level Version. The questionnaire items referred to positive vitality and available energy (for example, “I feel alive and vital”) without momentary anchors such as “right now.” After reverse coding, higher scores indicated greater energy depletion and lower available psychological energy (Cronbach’s α = 0.843).

Job crafting was measured using the 21-item scale developed by Tims et al. (2012), covering increasing structural job resources (5 items, Cronbach’s α = 0.875), increasing social job resources (6 items, Cronbach’s α = 0.808), increasing challenging job demands (5 items, Cronbach’s α = 0.812), and decreasing hindering job demands (5 items, Cronbach’s α = 0.832). A sample item is “I try to develop my capabilities.” The overall scale showed high internal consistency (Cronbach’s α = 0.920).

Work engagement was assessed using the 18-item scale developed by Kuok and Taormina (2017), covering cognitive, emotional, and physical engagement (6 items per dimension). A sample item is “My mind is often full of ideas about my work.” Reliability was high for the three dimensions (Cronbach’s α = 0.929, 0.960, and 0.969, respectively) and for the overall scale (Cronbach’s α = 0.970).

Mindfulness was measured using the 15-item Mindful Attention Awareness Scale (Brown and Ryan, 2003). Items were rated on a 6-point scale from 1 (almost always) to 6 (almost never), so higher scores reflected greater mindfulness. A sample item is “I could be experiencing some emotion and not be conscious of it until some time later” (Cronbach’s α = 0.900).

Control variables included gender, age, education level, and industry category. Industry was classified into ten categories, including information technology, electronics manufacturing, and finance.

A translation and back-translation procedure was adopted for all measurement items. The original items were translated into Chinese and reviewed by bilingual experts to resolve discrepancies, and then translated back into English. The wording and structure of the items were retained, and all items were scored according to the original scale guidelines.

### 3.2 Participants and sample

Participants were recruited from digitally intensive organizations in which information, communication, and computing technologies were embedded in daily operations (Bharadwaj et al., 2013). This recruitment context corresponds to the digitally mediated work context examined in this study: employees routinely coordinated tasks, communicated, and processed work information through multiple digital systems. The sample came from 12 medium-to-large enterprises in Shanghai, Guangzhou, and northern China, covering technology, finance, and advanced manufacturing. Participating organizations were identified through institutional contacts and professional networks associated with doctoral researchers and university alumni.

Data were collected through a three-wave survey design to reduce potential common method bias. Participants were matched across waves using unique digital identifiers. Wave 1, conducted from December 2022 to March 2023, collected demographic information, TO, and mindfulness. Wave 2, conducted from April to July 2023, measured energy depletion and job crafting. Wave 3, conducted from August to November 2023, assessed work engagement. Participation was voluntary at each wave, and respondents could choose whether to participate in subsequent waves. The final analytic sample was restricted to respondents who provided valid, matchable responses across all three waves.

Of the 1,000 questionnaires distributed at Wave 1, 765 valid responses were obtained (response rate = 76.5%). Among these respondents, 562 completed Wave 2 (retention rate = 73.5%), and 438 completed Wave 3 and could be matched across all three waves (retention rate from Wave 2 = 77.9%). The final matched sample consisted of 438 employees. Because the retained analytic dataset included only respondents who completed all three waves and provided valid, matchable responses, analyzable information on respondents who did not complete the full study was not retained under the data-collection plan. As a result, a formal comparison between retained and nonretained cases could not be conducted. Attrition-related selection bias is acknowledged in the limitations section.

In the final sample, 62.1% of participants were male, and 37.9% were female. Most participants were aged 30–50 years (46.6%), followed by 20–30 years (27.4%), and 50 years or above (26.0%). Most held at least a bachelor’s degree (73.7%).

### 3.3 Data analysis

Cases with more than 10% missing data were excluded. For the remaining observations, missing values were limited to approximately 2% and were handled using scale-level person-mean substitution. Skewness and kurtosis values fell within ±1.5, suggesting acceptable univariate normality.

We used several procedural remedies to reduce potential common method bias, including standardized instructions, time-lagged measurement, unique matching identifiers, reverse-coded items, and randomized item order. Common method variance was also assessed using Harman’s single-factor test with unrotated principal component analysis in SPSS 26.0 (Podsakoff et al., 2003). The first unrotated factor accounted for 31.36% of the total variance, below the commonly used 50% reference value. We treated this result as a diagnostic check rather than definitive evidence that common method bias was absent.

The analyses proceeded in two steps. First, confirmatory factor analysis (CFA) was conducted in Mplus 8.3 to examine the distinctiveness of the study constructs. Model fit was assessed using chi-square, CFI, TLI, and RMSEA, following conventional recommendations for measurement model evaluation (Hu and Bentler, 1999; Hair et al., 2019).

Second, after evaluating the measurement model, composite scores were computed by averaging the relevant items for each construct. The mediation and moderated mediation hypotheses were then tested in Mplus 8.3 using these composite scores as manifest variables (Muthén and Muthén, 1998–2017). Models were estimated with maximum likelihood using 5,000 bootstrap resamples. Indirect and conditional indirect effects were evaluated using bootstrapped confidence intervals, consistent with recommendations for mediation and moderated mediation analysis (Preacher et al., 2007; Preacher and Hayes, 2008). Predictors involved in interaction terms were grand-mean centered before product terms were created. Conditional effects were evaluated one standard deviation above and below the mean of mindfulness.

#### 3.3.1 Confirmatory factor analysis

The structural validity of the measurement model was assessed using confirmatory factor analysis (CFA). Based on theoretical considerations, a five-factor model was specified and compared with four-factor, three-factor, two-factor, and single-factor alternatives. As shown in Table 1, the five-factor model showed acceptable fit (χ^2^/df = 3.215, CFI = 0.916, TLI = 0.902, RMSEA = 0.051) and fit the data better than the alternative models, consistent with conventional fit-index guidelines (Hu and Bentler, 1999; Hair et al., 2019). Discriminant validity was further assessed using the heterotrait-monotrait ratio (HTMT; Henseler et al., 2015). HTMT values ranged from 0.291 to 0.862, below the 0.90 criterion. Multicollinearity was assessed using variance inflation factors (VIF), and all VIF values were below 5 (maximum VIF = 3.347). These results support the reliability and discriminant validity of the measures.

**Table 1:** Model comparison results for confirmatory factor analysis.

**Model**	***χ*** ^***2***^	***df***	***χ*** ^***2***^ ***/df***	**CFI**	**TLI**	**RMSEA**
Five-factor model (X, W, M1, M2, Y)	7288.985	2,267	3.215	0.916	0.902	0.051
Four factors model (X + W, M1, M2, Y)	8164.068	2,271	3.595	0.891	0.851	0.059
Three-factor model (X + M1 + M2, W, Y)	9055.397	2,274	3.982	0.765	0.712	0.083
Two-factor model (X + M1 + M2 + W, Y)	10930.887	2,276	4.803	0.687	0.631	0.086
One factor model (X + W + M1 + M2 + Y)	11531.832	2,277	5.064	0.559	0.505	0.086

X = TO; W = mindfulness; M1 = energy depletion; M2 = job crafting; Y = work engagement; χ^2^/df = normed chi square. Relative indexes: CFI = comparative fit index; TLI = Tucker-Lewis Index. Fit indexes for comparing nonnested models: RMSEA = root mean square error of approximation.

#### 3.3.2 Correlation analysis

**Figure 2:**
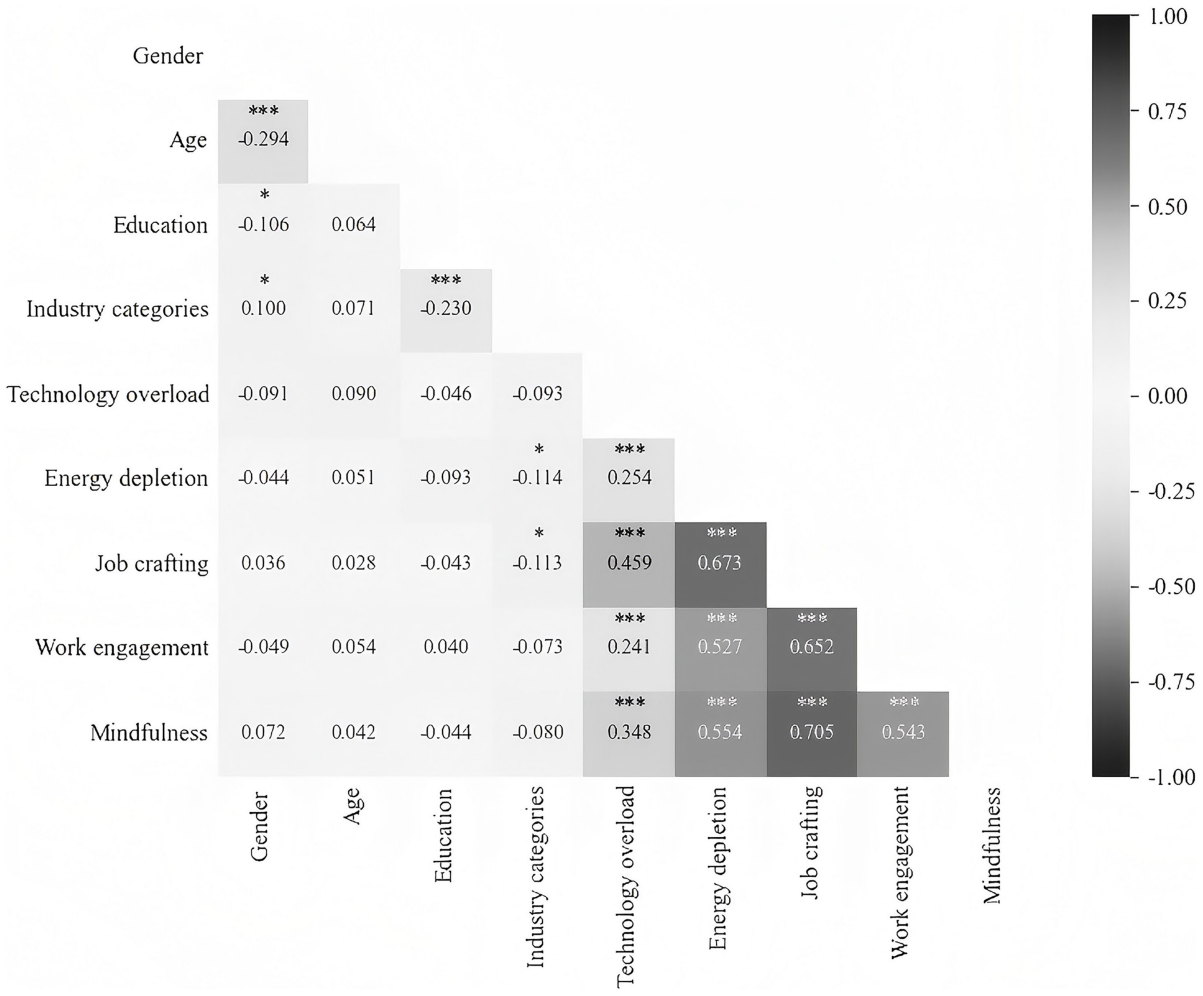
Correlation heatmap of all study variables. *p < 0.05; **p < 0.01; ***p < 0.001.

The correlations among key variables were examined using Pearson correlation coefficients. Technology overload was positively correlated with energy depletion (*r* = 0.254, *p* < 0.01), job crafting (*r* = 0.459, *p* < 0.01), and work engagement (*r* = 0.241, *p* < 0.01). Energy depletion was positively correlated with job crafting (*r* = 0.673, *p* < 0.01), and job crafting was positively correlated with work engagement (*r* = 0.652, *p* < 0.01). Figure 2 presents the correlation heatmap and provides a visual overview of the associations among the focal variables.

## 4 Results

### 4.1 Direct and mediated effects

The proposed model was estimated using Mplus 8.3, with unstandardized coefficients reported in Table 2. Technology overload was positively associated with energy depletion (*b* = 0.216, *p* < 0.001), supporting H1. Energy depletion was positively associated with job crafting (*b* = 0.438, *p* < 0.001), supporting H2. Job crafting was positively associated with work engagement (*b* = 0.724, *p* < 0.001), supporting H4. The direct path from technology overload to work engagement was negative but not statistically significant (*b* = −0.053, *p* = 0.072), suggesting that any association between technology overload and work engagement was more likely to operate through indirect pathways in this model.

**Table 2:** Structural path coefficients.

**Variables**	**Energy depletion**	**Job crafting**	**Work engagement**
Gender			−0.068 (0.042)
Age			0.001 (0.003)
Education			0.053* (0.026)
Industry			0.005 (0.006)
Technology overload	0.216*** (0.045)	0.193*** (0.020)	−0.053 (0.029)
Energy depletion		0.438*** (0.025)	0.143** (0.050)
Job crafting			0.724*** (0.076)

Entries are unstandardized coefficients with standard errors in parentheses. *p < 0.05; **p < 0.01; ***p < 0.001.

Bias-corrected bootstrapping was used to assess indirect effects. As shown in Table 3, the indirect effect of technology overload on job crafting through energy depletion was significant (estimate = 0.095, 95% CI [0.053, 0.134]), supporting H3. The indirect effect of energy depletion on work engagement through job crafting was also significant (estimate = 0.317, 95% CI [0.247, 0.393]), supporting H5. The sequential indirect effect of technology overload on work engagement through energy depletion and job crafting was significant as an additional indirect pathway (estimate = 0.068, 95% CI [0.038, 0.103]).

**Table 3:** Bootstrap estimates of direct and indirect effects.

**Pathway**	**Estimate**	**SE**	**95% CI lower**	**95% CI upper**
TO - > WE (direct)	−0.053	0.029	−0.111	0.005
TO - > ED - > JC (H3)	0.095***	0.021	0.053	0.134
ED - > JC - > WE (H5)	0.317***	0.037	0.247	0.393
TO - > ED - > WE	0.031*	0.013	0.010	0.060
TO - > JC - > WE	0.139***	0.022	0.099	0.185
TO - > ED - > JC - > WE	0.068***	0.016	0.038	0.103
Total indirect TO - > WE	0.239***	0.033	0.175	0.305

TO = technology overload; ED = energy depletion; JC = job crafting; WE = work engagement. CI = bias-corrected bootstrap confidence interval. *p < 0.05; **p < 0.01; ***p < 0.001.

### 4.2 Moderating effects

We examined the moderating role of mindfulness by testing two interaction effects: technology overload × mindfulness on energy depletion, and energy depletion × mindfulness on job crafting. Predictors involved in the interaction terms were grand-mean centered before product terms were created, and the same covariates used in the main model were included. Table 4 reports the results.

**Table 4:** Moderating effects of mindfulness on the structural model.

**Variables**	**Energy depletion (b, SE)**	**Job crafting (b, SE)**	**Work engagement (b, SE)**
Gender	−0.087 + (0.051)	0.044 (0.027)	−0.081 + (0.042)
Age	0.002 (0.003)	−0.001 (0.002)	0.000 (0.003)
Education	−0.066* (0.030)	0.017 (0.016)	0.052* (0.025)
Industry	−0.016 + (0.008)	−0.001 (0.004)	0.005 (0.006)
Technology overload	−0.004 (0.044)	0.130*** (0.020)	−0.059* (0.029)
Energy depletion		0.288*** (0.030)	0.121* (0.050)
Job crafting			0.613*** (0.090)
Mindfulness	0.613*** (0.054)	0.352*** (0.037)	0.173** (0.062)
Mindfulness x technology overload	0.154** (0.058)		
Mindfulness x energy depletion		0.071 (0.037)	

Entries are unstandardized coefficients with standard errors in parentheses. TO = technology overload; ED = energy depletion; JC = job crafting; MF = mindfulness. Predictors involved in interaction terms were grand-mean centered; therefore, main effects in the interaction model represent conditional effects when the interacting variable is at its mean. +*p* < 0.10; *p < 0.05; **p < 0.01; ***p < 0.001.

#### 4.2.1 Moderation of the technology overload-energy depletion relationship

The interaction between technology overload and mindfulness was positively associated with energy depletion (*b* = 0.154, *p* = 0.008, 95% CI [0.036, 0.266]). Simple slope analysis indicated that the association between technology overload and energy depletion was positive and statistically significant when mindfulness was high (*b* = 0.072, *p* = 0.034, 95% CI [0.007, 0.139]), but nonsignificant when mindfulness was low (*b* = −0.080, *p* = 0.225, 95% CI [−0.204, 0.055]). These results support H6a.

#### 4.2.2 Moderation of the energy depletion-job crafting relationship

The interaction between energy depletion and mindfulness was positive but did not reach conventional statistical significance (*b* = 0.071, *p* = 0.053, 95% CI [−0.002, 0.142]). Simple slopes were positive at both low mindfulness (*b* = 0.253, p < 0.001, 95% CI [0.181, 0.326]) and high mindfulness (*b* = 0.323, *p* < 0.001, 95% CI [0.256, 0.382]), with a numerically steeper slope at higher mindfulness. Thus, H6b was not clearly supported, although the pattern was in the expected direction.

#### 4.2.3 Conditional indirect effects

Conditional indirect effects were examined at one standard deviation above and below the mean of mindfulness. As shown in Table 5, the indirect effect of technology overload on job crafting through energy depletion was not significant at low mindfulness (estimate = −0.020, 95% CI [−0.057, 0.012]) but was significant at high mindfulness (estimate = 0.023, 95% CI [0.002, 0.047]). The difference between these conditional indirect effects was significant (*Δ* = 0.044, 95% CI [0.015, 0.077]), supporting H7a.

**Table 5:** Conditional indirect effects of mindfulness.

Pathway	Level	Indirect effect	SE	95% CI lower	95% CI upper
TO - > ED - > JC (H7a)	Low MF	−0.020	0.017	−0.057	0.012
	High MF	0.023*	0.011	0.002	0.047
	Delta	0.044**	0.016	0.015	0.077
ED - > JC - > WE (H7b)	Low MF	0.155***	0.032	0.100	0.225
	High MF	0.198***	0.034	0.138	0.271
	Delta	0.043	0.023	0.000	0.090
TO - > ED - > JC - > WE	Low MF	−0.012	0.011	−0.037	0.007
	High MF	0.014*	0.007	0.002	0.031
	Delta	0.027*	0.011	0.010	0.052

TO = technology overload; ED = energy depletion; JC = job crafting; WE = work engagement; MF = mindfulness. CI = bias-corrected bootstrap confidence interval. *p < 0.05; **p < 0.01; ***p < 0.001.

For H7b, the indirect effect of energy depletion on work engagement through job crafting was significant at both low mindfulness (estimate = 0.155, 95% CI [0.100, 0.225]) and high mindfulness (estimate = 0.198, 95% CI [0.138, 0.271]). The difference between these conditional indirect effects was positive but marginal (Δ = 0.043, *p* = 0.063, 95% CI [0.000, 0.090]). Therefore, H7b received only limited evidence and should be interpreted cautiously. The sequential indirect effect of technology overload on work engagement through energy depletion and job crafting was significant at high mindfulness (estimate = 0.014, 95% CI [0.002, 0.031]) but not at low mindfulness (estimate = −0.012, 95% CI [−0.037, 0.007]). The difference between these conditional sequential indirect effects was significant (Δ = 0.027, 95% CI [0.010, 0.052]).

**Figure 3:**
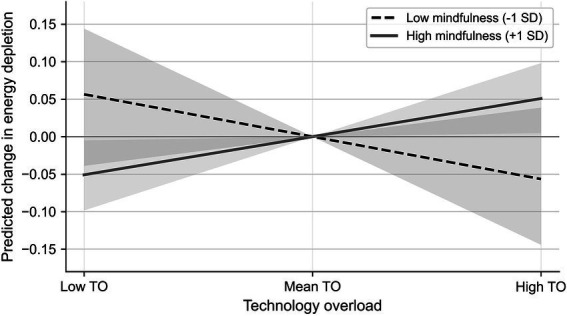
Simple slopes of technology overload (TO) on energy depletion at different levels of mindfulness. The solid line represents high mindfulness (+1 SD) and shows a significant positive slope (*p* < 0.05), whereas the dashed line represents low mindfulness (−1 SD) and shows a non-significant slope (*p* > 0.05). Shaded areas represent 95% confidence intervals.

**Figure 4:**
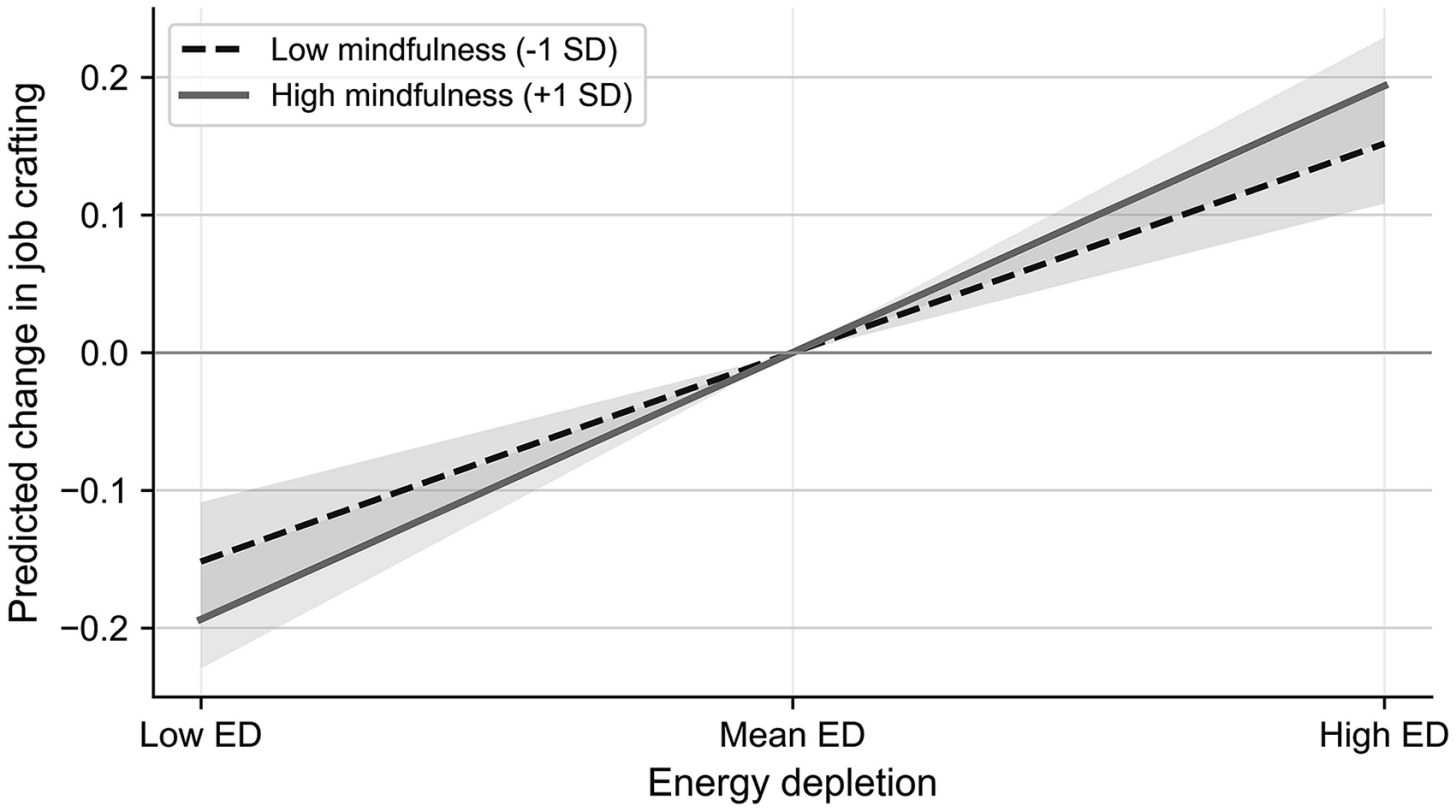
Simple slopes of energy depletion on job crafting at different levels of mindfulness. Both simple slopes were positive. The interaction term approached, but did not reach, conventional significance and should be interpreted cautiously. Shaded areas represent 95% confidence intervals.

Taken together, these findings suggest that mindfulness showed clearer evidence of moderating the link between technology overload and energy depletion, whereas evidence for its role in the pathway from energy depletion to job crafting was weaker (Figures 3, 4).

## 5 Discussion

These findings point to an indirect link between TO and work engagement through energy depletion and subsequent job crafting. The nonsignificant direct effect, together with the significant indirect pathways, suggests that this relationship is better understood through intervening psychological and behavioral processes. TO appears to consume internal resources, and employees may respond to this pressure by adjusting their work behavior to cope with ongoing digital demands. Importantly, the positive indirect path should not be interpreted as evidence that TO is beneficial. It indicates that some employees may attempt to adapt despite overload, not because overload itself is healthy or desirable.

Although we did not directly assess whether TO was appraised as a challenge or a hindrance, our interpretation is consistent with appraisal-based theoretical classifications. TO shares some features with traditional challenge demands such as workload and time pressure because it involves high requirements and performance-related expectations (Tarafdar et al., 2015). At the same time, TO should not be treated as uniformly beneficial, uniformly challenging, or equivalent to the digital workplace itself. It is better understood as a perceived overload state that emerges from the interaction between digital systems, organizational expectations, and available resources. The observed sequence suggests that TO may involve resource costs while also being followed by compensatory adjustment under certain conditions. This distinction addresses the apparent tension between TO as a challenge-like demand and TO as a source of energy depletion: depletion remains a cost of overload, whereas job crafting is one possible response to that cost.

Mindfulness showed a more nuanced moderating role than originally expected. The evidence was clearer for the path from TO to energy depletion: employees with higher mindfulness showed a stronger association between TO and energy depletion. This finding suggests that mindfulness may increase awareness of technology-related demands and make early signs of resource depletion more salient. Mindfulness therefore does not appear to function simply as a buffer against TO. It may also heighten sensitivity to internal resource demands.

The evidence for the moderating role of mindfulness in the relationship between energy depletion and job crafting was not clear. Although the interaction was positive and in the expected direction, it did not reach conventional levels of statistical significance. One possible explanation is that job crafting requires more than awareness and self-regulation. Employees may also need autonomy, psychological safety, supportive supervision, sufficient discretion, and decision latitude to adjust tasks and seek resources (Karasek, 1979). Mindfulness may help employees recognize depletion, but it may not consistently translate depletion into job crafting when contextual support is limited. The evidence for the corresponding conditional indirect effect from energy depletion to work engagement through job crafting was limited and should be interpreted cautiously.

The organizational context may also shape these processes. The study was conducted in Chinese organizations, where work engagement may be strongly emphasized and withdrawal behavior may be discouraged (Wang and Yi, 2012). Continuous responsiveness to digital demands may be interpreted as diligence or professional reliability, even when it reflects underlying pressure. Because this study did not directly measure cultural values, this interpretation remains tentative. Future research could examine how individual-level cultural values influence the appraisal and outcomes of digital job demands.

### 5.1 Theoretical implications

Technology overload is often defined as a negative factor that impairs psychological functioning and reduces performance (Tarafdar et al., 2015), implying that individuals respond to TO in a relatively uniform manner. However, responses to digital demands are shaped by the social and organizational setting in which work is carried out. Bobillier-Chaumon (2021) argues that digital technologies transform work activity by changing how tasks are prescribed, interrupted, monitored, and negotiated with others. In environments characterized by centralized authority and relationally grounded expectations, digital demands also carry additional social meaning (Cui et al., 2021). For instance, expectations of constant availability may signal both workload intensity and role significance, as responsiveness is often associated with professional dedication. Cross-cultural research indicates that such expectations are interpreted differently across contexts, influencing whether they are experienced as pressure or as value affirmation (Lee et al., 2021; Tran et al., 2022). These differences highlight the role of cultural meaning systems and work-activity conditions in shaping how job demands are appraised and extend CHDF by situating these processes within specific social contexts (Podsakoff et al., 2023).

The work environment shaped by cultural norms may influence how individuals interpret energy depletion. In the Chinese context, sustained effort is often socially reinforced and embedded in role expectations. As a result, energy depletion does not necessarily lead to immediate psychological withdrawal (Tims et al., 2012; Zhang and Parker, 2019). Instead, it may prompt individuals to adjust how they engage in their work, thereby maintaining involvement and regaining a sense of control. One form of this adjustment is job crafting (Wrzesniewski and Dutton, 2001; Tims et al., 2013). However, this interpretation should not minimize the negative meaning of depletion. Persistent exhaustion, cynicism, and reduced professional efficacy can undermine employees’ health and work functioning over time (Maslach et al., 2001; Schaufeli et al., 2002). Job crafting in this context therefore reflects a culturally shaped response to strain that may help maintain engagement, but it should be understood as a compensatory response rather than a sign that depletion is harmless.

The relationship between energy depletion and job crafting seems to depend on both the individual’ s ability to regulate internal stress and the work context that enables or constrains action. Mindfulness appears as a factor at different stages, exerting varying effects across the process of resource loss and adjustment. In the early stage, individuals with high mindfulness report a stronger response to internal strain signals, reflecting greater sensitivity to such signals (Allen et al., 2021). This awareness may strengthen the perceived gap between internal states and external demands. Prior research suggests that mindfulness may support role adjustment by helping employees direct their attention and effort toward crafting tasks or interactions (Glomb et al., 2011). However, the present findings suggest that this later-stage role depends more on contextual support than expected. These findings challenge the view that mindfulness consistently reduces stress, instead pointing to its stage-dependent and context-dependent effects. These dynamics are broadly consistent with MAT, which suggests that monitoring enhances awareness, while acceptance enables effective regulation (Lindsay and Creswell, 2017). This dual mechanism may be particularly relevant in high-context cultures, where individuals are expected to interpret implicit demands and maintain relational harmony under pressure (Spencer-Rodgers et al., 2010). This stage-based process provides a more differentiated explanation of how self-regulation unfolds in TO, while also showing why self-regulation alone may be insufficient when employees lack autonomy or organizational flexibility.

The findings suggest that TO may be followed by active adjustment in some contexts. From a COR perspective, this process begins with energy depletion under perceived stress. Depletion may signal a need to adjust how work is managed, rather than leading directly to withdrawal. When employees have regulatory capacity and enough discretion over their work, they may invest in job crafting to regain some control over work demands. Cultural expectations about persistence and responsibility may also shape whether such effort is sustained. In this sense, job crafting may help explain how employees maintain work engagement despite ongoing digital demands. Mindfulness appears to shape this process differently across stages. Greater awareness of fatigue may increase perceived resource costs at an early stage, while the evidence that mindfulness supports later behavioral adjustment was weaker in the present study. Consistent with CHDF, TO may resemble a challenge-like demand under certain conditions, but it remains a source of resource loss and a possible occupational health risk. TO should therefore be understood as a context-dependent digital work demand, not as inherently positive or negative. Its effects may depend on employees’ awareness of strain, the way digital tools shape everyday work, and the social expectations attached to continued effort.

### 5.2 Practical implications

TO is difficult to eliminate because employees increasingly rely on multiple information, communication, and computing systems in daily work. The speed and intensity of digital information often exceed employees’ ability to keep up, making TO a recurring challenge. Prior research has shown that technostress can undermine well-being and performance, while its outcomes may also depend on employees’ appraisals, coping resources, and organizational support (Ragu-Nathan et al., 2008; Tarafdar et al., 2015; Chandra et al., 2019; Zhao et al., 2020; Castillo et al., 2023). Organizational responses should go beyond helping employees adapt after overload has occurred. Organizations also need to reduce avoidable digital demands and treat energy depletion as an early warning sign of resource loss. When digital demands accumulate, managers can use signs of fatigue to review communication expectations, reduce avoidable interruptions, and protect employees’ recovery time. Organizations can also support technology adaptation by embedding technical guidance into daily work and involving employees in the design or refinement of digital systems (Beaudry and Pinsonneault, 2005; Pirkkalainen et al., 2019). Employees are more likely to treat TO as manageable when they have clear norms, usable support, and some discretion over how digital work is organized.

A second implication concerns job crafting. Energy depletion is often associated with withdrawal, but our findings suggest that it may also precede job crafting as employees try to adjust work before disengaging from it. This interpretation is consistent with evidence that employees may craft their jobs even under difficult work conditions (Wang et al., 2018), and with research showing that job crafting can help sustain engagement through adjustments to job demands and resources (Tims et al., 2013; Petrou et al., 2015; Zhang and Parker, 2019). Organizations can support this process by giving employees discretion and decision latitude over how they organize digital tasks and by making feedback or peer support easier to access (Karasek, 1979). They should also allow employees to reduce unnecessary hindering demands where possible. Practical steps include simplifying digital workflows, reducing avoidable interruptions, improving system usability, and integrating training resources into daily work instead of adding them as extra demands.

Finally, mindfulness-based practices should be implemented carefully. Mindfulness may support attention regulation and stress management, but its effects depend on how it is introduced and whether it fits the work context (Hülsheger et al., 2013; Reb et al., 2015; Krick et al., 2023). Organizations should avoid using mindfulness programs as a substitute for better work design, healthier digital workload management, or stronger occupational health protections. Programs that lack a clear purpose or do not address actual job demands may be perceived as superficial. Mindfulness initiatives are more likely to be useful when they are voluntary, evidence-based, and embedded in broader development or recovery-oriented systems. These practices are most likely to support adaptive responses to TO when they are paired with clearer communication norms, more manageable technology use, and real opportunities to adjust work activity.

### 5.3 Limitations and future research

First, although the three-wave design helps reduce common method concerns, it does not establish causality or capture how resource dynamics unfold in real time. Longitudinal, diary, or experience-sampling designs could better examine how TO, energy depletion, job crafting, and work engagement fluctuate in daily digital work (Gabel-Shemueli et al., 2019). The reliance on self-reported data may also introduce common method bias or socially desirable responding, despite the use of time-lagged measurement and anonymous matching (Podsakoff et al., 2003). Future studies could incorporate supervisor ratings, peer reports, or behavioral indicators of digital work. In addition, the final analytic sample was restricted to respondents with valid, matchable responses across all three waves. Because analyzable information on non-completers was not retained under the data-collection plan, we could not formally compare retained and nonretained cases. Attrition-related selection bias therefore cannot be fully ruled out.

The sample was drawn from Chinese organizations and mainly included employees with undergraduate or higher education. This may limit generalizability to other occupational or cultural contexts. Cultural norms around performance and responsibility may also shape how employees report job crafting, work engagement, and mindfulness (Hofstede, 2011; Hu et al., 2014; Tran et al., 2022). More diverse and measurement-equivalent cross-cultural samples would help clarify whether the relationships among TO, energy depletion, job crafting, and work engagement differ across contexts.

A further limitation concerns how participants may have interpreted the focal constructs. This study treated TO as a challenge-like digital overload state, but employees may appraise TO differently depending on available resources, occupational context, decision latitude, and organizational support (Karasek, 1979; Lazarus and Folkman, 1984; Lepine et al., 2005; Zhao et al., 2020). Later work could examine real-time appraisals of TO and clarify when digital demands are experienced as challenges, hindrances, or threats. It could also examine how prescribed work, actual work activity, and organizational flexibility shape employees’ ability to craft their jobs in response to overload. Most participants had not received formal mindfulness training and may have interpreted mindfulness as general attention. Future studies could compare employees with different levels of mindfulness training or practice experience.

Finally, this study focused on job crafting as an adaptive response, but energy depletion may also lead to withdrawal, avoidance, burnout-related exhaustion, or reduced engagement. Future research could examine why some employees respond to TO by crafting their jobs, whereas others disengage or experience longer-term health impairment. It would also be useful to distinguish among job crafting forms, as seeking resources, taking on challenges, and reducing hindering demands may have different implications for recovery and engagement (Tims et al., 2012; Petrou et al., 2015; Zhang and Parker, 2019). Such work would help clarify when adaptation is sustainable and when it masks continuing resource loss.

## 6 Conclusion

To understand employees’ reactions to TO, it is necessary to pay closer attention to the conditions under which overload produces resource loss and when employees are still able to respond adaptively. The findings suggest that these responses depend on individuals’ regulatory resources and how the organizational environment shapes the interpretation of demands. This does not mean that overload is beneficial in itself. On the contrary, the results point to a more complex process in which psychological capacity, work activity, decision latitude, and cultural meaning shape how individuals experience and manage digital strain. A resource-based and appraisal-based perspective helps explain why the same digital demands may produce different experiences of strain. Employees’ responses depend not only on the demands themselves, but also on how they appraise the situation, regulate their effort, and use available support at work.

## Data Availability

The raw data supporting the conclusions of this article will be made available by the authors, without undue reservation.
